# Pharmacokinetics of Tylvalosin Following Intravenous or Oral Administration at Different Doses in Broiler Chickens

**DOI:** 10.3390/vetsci12020118

**Published:** 2025-02-02

**Authors:** Zeyu Wen, Sumeng Chen, Jinyan Meng, Qinyao Wu, Runlin Yu, Nuoyu Xu, Jingyuan Kong, Lu Zhang, Xingyuan Cao

**Affiliations:** 1 Department of Veterinary Pharmacology and Toxicology, College of Veterinary Medicine, China Agricultural University, Beijing 100193, China; wenzy1124@163.com (Z.W.); for0ever@126.com (S.C.); jinyanmeng00@outlook.com (J.M.); wuqinyao7711@163.com (Q.W.); yurunliny@163.com (R.Y.); 2019305010115@cau.edu.cn (N.X.); jingyuankong@outlook.com (J.K.); violetlily209@163.com (L.Z.); 2 Key Laboratory of Detection for Veterinary Drug Residues and Illegal Additives, Ministry of Agriculture and Rural Affairs of the People’s Republic of China, Beijing 100193, China

**Keywords:** tylvalosin, pharmacokinetics, chickens

## Abstract

Tylvalosin is a macrolide antimicrobial with antibacterial activity against Gram-positive bacteria, some Gram-negative organisms, and mycoplasma. It is used to treat respiratory and enteric bacterial infections in swine and poultry. In this study, we aimed to investigate the pharmacokinetic changes in tylvalosin following its intravenous or oral administration at doses of 5, 10, and 25 mg/kg in broiler chickens. Forty-eight broiler chickens were included in the study. The plasma concentrations of tylvalosin were measured by using ultra-performance liquid chromatography–tandem mass spectrometry (UPLC-MS/MS), and its pharmacokinetic parameters were evaluated by using both non-compartmental and compartmental analyses. The linear mixed-effects model revealed no dose proportionality within the 5–25 mg/kg range for either administration route. Based on pharmacokinetic data from a single oral dose, this study simulated a multiple-dose regimen of tylvalosin (25 mg/kg), demonstrating that a 6-hour dosing interval reaches a steady state after the fourth dose. Furthermore, the results show that the absolute bioavailability of tylvalosin after oral administration in chickens was relatively low, with values of 5.92%, 3.56%, and 3.04% for the doses of 5, 10, and 25 mg/kg, respectively. Further studies are required to significantly improve the oral bioavailability of tylvalosin and similar compounds through formulation optimization.

## Simple Summary

This study aimed to investigate the pharmacokinetics of tylvalosin following oral and intravenous administration at three different doses in broiler chickens. Forty-eight healthy chickens were selected for this study and maintained in a controlled environment throughout the experiment, with no adverse effects observed. Each chicken received a single dose of tylvalosin via its respective route of administration. Blood samples were collected at predetermined time points and analyzed using ultra-performance liquid chromatography–tandem mass spectrometry (UPLC-MS/MS). Pharmacokinetic parameters were calculated using professional software, and a linear mixed-effects model was applied. The results revealed a lack of dose proportionality within the 5–25 mg/kg range for both administration routes. Additionally, this study simulated a multiple-dose regimen of tylvalosin (25 mg/kg) based on pharmacokinetic data from a single oral dose, with results showing that a 6-hour dosing interval achieves a steady state after the fourth dose. Furthermore, the study highlighted the need to enhance the oral bioavailability of tylvalosin due to its low absolute bioavailability.

## 1. Introduction

*Mycoplasma gallisepticum* (MG) and *Mycoplasma synoviae* (MS) are widely recognized as major infectious agents in poultry, causing significant economic losses [1]. These pathogens are the primary etiological agents responsible for respiratory diseases and infectious synovitis in chickens and turkeys. The results of chronic respiratory disease include reduced growth rates, decreased egg production and hatchability, and significant decreases in carcass quality [2,3,4,5,6]. It is of the utmost importance to be able to diagnose infections and to treat these species in poultry effectively, given that they have two routes of transmission: horizontal and vertical [7,8].

At the present time, antimicrobial therapy represents the most rapid and effective approach for the treatment of mycoplasma infections in poultry farms. MG and MS have demonstrated in vitro and in vivo susceptibility to a number of classes of antimicrobials, including macrolides (tylosin, tilmicosin, tylvalosin, erythromycin, and spiramycin), tetracyclines (oxytetracycline, chlortetracycline, and doxycycline), and quinoloness (enrofloxacin) [5,6,9,10,11]. Macrolide antibiotics and their semi-synthetic derivatives, in particular those which exhibit potent antibacterial activity against mycoplasma, such as tylosin, tylvalosin, and tilmicosin, have shown promising efficacy against MS.

In a previous study, the use of both conventional broth microdilution and quantitative real-time polymerase chain reaction assays for the evaluation of the in vitro efficacy of various antimicrobials against field MG and MS isolates recovered from chicken and turkey flocks revealed that tylvalosin was the most active antibiotic against the MG and MS isolates with the lowest MIC values (0.015 to 0.03 µg/mL) [12]. Furthermore, other researchers reported an MIC_50_ value of 0.0098 µg/mL for tylvalosin, which makes it more effective than tylosin, tiamulin, doxycycline, oxytetracycline, tilmicosin, and lincomycin–spectinomycin [11]. This finding underscores the superior efficacy of tylvalosin among the tested antimicrobials and highlights its potential importance in controlling mycoplasma infections in chickens and turkeys.

Tylvalosin is a novel veterinary macrolide antibiotic with a 16-membered lactone ring, synthesized by changing the 3-acetyl-40-isovaleryl group into acetylisovaleryl tylosin tartrate group in tylosin [13,14]. It exhibits antibacterial activity against Gram-positive bacteria (e.g., *Staphylococcus*, *Micrococcus*, *Microbacterium*, *Bacillus*, *Corynebacterium*, *Aerococcus*, *Arthrobacter and Streptococcus*, *Campylobacter*, *Enterococcus*, and *Clostridia*), certain Gram-negative organisms, and mycoplasma by inhibiting bacterial protein synthesis through irreversible binding to the 50S ribosomal subunit of susceptible bacteria [15]. Due to its efficacy and safety in treating crucial poultry pathogens, such as *Mycoplasma* spp., *Brachyspira pilosicoli*, *Brachyspira hyodysenteriae*, *Clostridium* spp., and *Bacteroides* spp., tylvalosin has garnered considerable attention [10,16]. Furthermore, it has demonstrated potent anti-inflammatory, antioxidant, and immunomodulatory effects on porcine leukocytes and is widely used in swine for the treatment of porcine proliferative enteritis, swine enzootic pneumonia, and swine dysentery [17]. As a result, it has become one of the most employed agents in the swine industry for the treatment of Mycoplasma hyopneumoniae infections.

Tylvalosin, a widely used antibiotic, has been extensively studied in pigs and various poultry species [18,19,20]. To date, several pharmacokinetic studies on tylvalosin in chickens have been conducted. E.A. compared the relative bioavailability and pharmacokinetics of two commercially available oral formulations of tylvalosin, administered as a single oral dose (25 mg/kg) specifically designed for broiler chickens. The plasma concentrations for both formulations remained relatively high for at least 3 h after administration [20]. A.M. evaluated the pharmacokinetics of tylvalosin following oral, intramuscular, and intravenous administration and further investigated its bioavailability in healthy turkeys [13,15]. The results demonstrated that tylvalosin was rapidly absorbed and distributed to turkey tissues following oral administration. Research indicates that the pharmacokinetic (PK) characteristics of tylvalosin can be significantly influenced by administration route, dosage level, species, and formulation differences, ultimately affecting its efficacy and safety. However, comprehensive comparative analyses across different administration routes and dosage levels remain limited.

Considering the above facts, in this study, we aimed to systematically evaluate the pharmacokinetic profiles and bioavailability of tylvalosin in broiler chickens, comparing intravenous and oral administration at three dosage levels. The findings are expected to provide scientific evidence for optimizing formulation development and enhancing the therapeutic application of tylvalosin in the treatment of various diseases.

## 2. Materials and Methods

### 2.1. Chemicals and Reagents

Tylvalosin (tartrate) standard (CAS 63428-13-7; purity, 98.77%) was provided by MedChemExpress (Monmouth Junction, NJ, USA), Acetylisovaleryltylosin Tartrate (CAS 63428-1307) was provided by Widely (Hubei, China), and Tylvalosin Tartrate Soluble Powder was provided by Qilu Animal Health Products Co., Ltd. (Shandong, China). Each gram of powder contained 250 mg of Tylvalosin (as Tylvalosin Tartrate) as the active substance. HPLC-grade acetonitrile (ACN) and formic acid (FA) were provided by Thermo Fisher Scientific (Macquarie Park, NSW, Australia). Water was purified with a Milli-Q water purification system (Merck Millipore, Burlington, MA, USA).

### 2.2. Experimental Broiler Chickens

A total of 48 (24 males and 24 females for the PK study) apparently healthy 5–6-week-old broiler chickens weighing between 2.2 and 3.4 kg were obtained from the Laboratory Animal Center of China Agricultural University. Prior to enrolling in the study, all animals were acclimatized for 2 weeks in a controlled environment with a 12-hour light/dark cycle, maintaining an ambient temperature of 23 ± 2 °C and a relative humidity of 55 ± 10%.

The experimental protocol was ethically approved by the Animal Breeding and Use Committee of China Agricultural University (AW61214202-2-03) (Beijing, China).

### 2.3. Drugs Administration and Study Design

Before the administration of tylvalosin, the effective concentrations of tylvalosin in the product were quantified using an ultra-performance liquid chromatography–tandem mass spectrometer (UPLC-MS/MS; provided by Waters, Milford, CT, USA), confirming that the concentrations met the required standards. Prior to administration, the required amount of Tylvalosin Tartrate was weighed and dissolved in the appropriate solvent. For intravenous injection, the Acetylisovaleryltylosin Tartrate was dissolved in sterile saline for injection, followed by sonication to ensure uniform mixing. For oral administration, the required amount of Tylvalosin Tartrate Soluble Powder was dissolved in pure water, and sonication was also used to achieve complete homogeneity. Each animal in this study was given the drug only once according to its weight.

The 48 healthy broiler chickens were allocated into two groups of 24 each; the first group received a single intravenous dose (administered via the wing vein), while the second group was administered a single oral dose (via the intra-crop route). Each group was further subdivided into three dosage subgroups, consisting of 8 chickens each, with doses of 5, 10, and 25 mg/kg body weight. The experimental chickens were fasted for 12 h prior to administration but had free access to water. Each chicken was weighed immediately prior to drug administration on the first day of treatment.

Approximately 1.0–1.5 mL of whole-blood samples were collected from the wing vein into heparinized tubes before (time 0 h) and 0.083 (intravenous administration only), 0.25, 0.5, 1, 1.5, 2, 2.5, 3, 4, 6, 8, 10, 12, and 24 h after the administration of the drug. Afterward, the blood samples were centrifuged for 10 min at 6000 rpm, and plasma was aspirated and stored at −20 °C until analysis.

### 2.4. Sample Preparation

The frozen plasma samples were thawed at room temperature before sample preparation for about 15 min and vortexed, and 100 μL from each was accurately transferred to Eppendorf centrifuge tubes. A volume of 300 μL of acetonitrile was added to the tubes to precipitate the proteins, vortexed and mixed for 5 min, and then centrifuged at 4 °C and 10,000 rpm for 10 min. The supernatant was passed through 0.22 μm organic microporous filter membranes, vortexed, and mixed well, and 10 μL of the supernatant was used for UPLC-MS/MS analysis.

### 2.5. UPLC-MS/MS Conditions

Multiple-reaction monitoring (MRM) in positive-ion electrospray (ESI) mode in the UPLC-MS/MS was performed for drug quantification in this analytical process. The plasma extract samples were injected and separated at 0.3 mL/min in a column (ACQUITY UPLC HSS T3 Column, 1.8 µm, 2.1 mm × 100 mm) at 40 °C with two mobile phases: (A) 0.1% formic acid aqueous solution and (B) 0.1% formic acid acetonitrile. These two mobile phases were delivered according to the following program: 10–50% of B, 0–2.0 min; 50–90% of B, 2.0–2.5 min; 90% of B, 2.5–3.5 min; 90–10% of B, 3.5–5.0 min; 10% of B, 5.0–6.0 min.

### 2.6. Preparation of Standards

A stock solution of tylvalosin was prepared by accurately weighing 10.12 mg of tylvalosin standard and then dissolving it in 10 mL of acetonitrile to produce 1 mg/mL stock solution, which was stored at −20 °C. This solution was used to prepare calibration curves and quality control parameters (QCs) for method validation in the following: selectivity, precision and accuracy, and stability. Working solutions of the standard, ranging from 0.01 to 10 ug/mL, were freshly prepared by serially diluting the stock solution with acetonitrile for each use. A volume of 10 µL of each of the working solutions was added to 90 µL of blank chicken plasma (for total of 100 µL) to prepare the final calibration standards from 1 to 1000 ng/mL. Three concentrations of tylvalosin, which served as QCs at 2, 400, and 800 ng/mL, were also prepared in blank plasma and subsequently stored at −20 °C.

### 2.7. Method Validation

Calibration curves of tylvalosin were prepared over three consecutive days, demonstrating satisfactory linearity within the concentration range of 1 to 1000 ng/mL (r^2^ > 0.99). The selectivity of this method was assessed by injecting six blank plasma samples from different chickens following the previously described sample preparation, and no interfering peaks were observed in the tylvalosin region. The lower limit of quantification (LLOQ) for tylvalosin in plasma was 1 ng/mL. Intra- and inter-batch precision and accuracy were evaluated within a single day and across three consecutive days by using the LLOQ and QC samples at concentrations of 1, 2, 400, and 800 ng/mL. The results indicate that the intra- and inter-batch precision of the QC samples remained within ±15% (ranging from 2.85% to 11.19%), while the precision of the LLOQ samples was maintained within ±20% (ranging from 6.31 to 10.11%). The stability of the QC samples at room temperature (25 °C) and that of the automatic sampler at 15 °C were evaluated over a 24-hour period, demonstrating that both conditions remained stable.

The method validation in this study was based on the “Technical Guideline for Method Validation of Quantitative Analysis of Biological Samples”.

### 2.8. Data Analysis

Blood concentrations of tylvalosin were analyzed by using the established method, and the pharmacokinetic parameters were calculated with non-compartmental and compartmental analyses in WinNonlin software (WinNolin 8.3.4 Certara; Pharsight, Mountain View, CA, USA) and expressed as means ± standard deviations (SDs); a power model and one-way ANOVA tests were applied. The difference was significant when the *p*-value was smaller than 0.05.

Furthermore, computational simulations were calculated for the multiple-dose oral administrations of tylvalosin at a dosage of 25 mg/kg, with dosing intervals of 5, 6, and 7 h, respectively.

## 3. Results

Following the administration of three distinct doses to both the oral and intravenous groups, all the chickens exhibited tolerance to tylvalosin. No adverse effects, such as gastrointestinal disturbances, lethargy, or changes in behavior, were observed in any of the chickens during the 24-hour study period.

### 3.1. After Intravenous Injection

**Figure 1:**
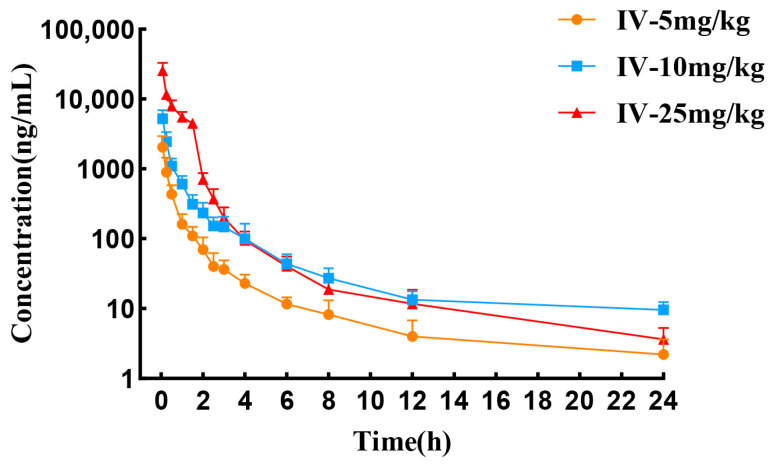
Semi-logarithmic plasma concentration–time curves following intravenous administration of tylvalosin at 5, 10, and 25 mg/kg doses in chickens (mean ± SD, *n* = 8).

The mean plasma concentration–time curves and pharmacokinetic parameters of tylvalosin following intravenous administration at doses of 5, 10, and 25 mg/kg in chickens are presented in Figure 1 and Table 1, respectively. Tylvalosin was detectable at all three doses up to 24 h. After intravenous administration at doses of 5, 10, and 25 mg/kg, the initial time of tylvalosin plasma concentrations were 2.05 ± 0.88, 5.21 ± 1.71, and 25.44 ± 7.43 μg/mL, respectively. The values of area under concentration/time curve from 0 to last point (AUC_last_) increased proportionally in the 5–10 mg/kg dose range (1.09 ± 0.38 and 3.18 ±0.86 h∗ug/mL, respectively). The elimination half-life (T_1/2λz_) at the dose of 25 mg/kg was more than that at other doses, indicating that the elimination process was slower at higher doses.

The clearance and volume of distribution of tylvalosin were used as initial values to estimate the parameters of the compartmental model. The secondary parameters are presented in Table 2. A two-compartment model was applied to describe the pharmacokinetics of tylvalosin in chickens following intravenous administration. The results demonstrate that the drug was rapidly distributed across the three different doses, with a distribution half-life (t_1/2α_) of approximately 0.12 h. As the dose increased, the mean elimination half-life (t_1/2β_) decreased within the 5–25 mg/kg dose range, measuring 1.83 ± 2.35, 0.86 ± 0.30, and 0.63 ± 0.21 h, respectively. In the low and medium intravenous groups, the apparent volume of distribution (Vc) of tylvalosin in the central compartment was relatively low (1.73 ± 1.37 and 1.42 ± 0.93 L/kg, respectively) compared with the apparent volume of distribution in the peripheral compartment (V2) (3.24 ± 2.55 and 1.93 ± 1.14 L/kg, respectively) and the total volume of distribution at steady state (Vss) (4.97 ± 3.60 and 3.35 ± 1.99 L/kg, respectively).

### 3.2. After Oral Injection

**Figure 2:**
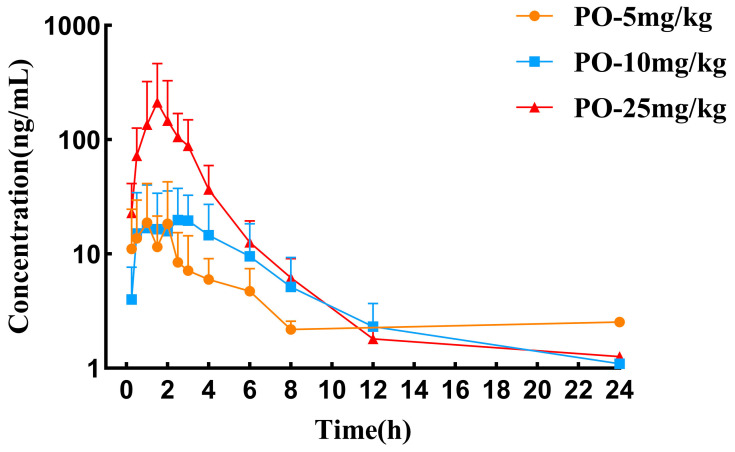
Semi-logarithmic plasma concentration–time curves following oral administration of tylvalosin at 5, 10, and 25 mg/kg doses in chickens (mean ± SD, *n* = 8).

The mean plasma concentration–time curves and the pharmacokinetic parameters of tylvalosin following its oral administration to chickens at doses of 5, 10, and 25 mg/kg are presented in Figure 2 and summarized in Table 3. Tylvalosin was undetectable in some samples after 12 h. Following oral administration, the drug reached its peak concentration within 3 h at all three doses. The maximum plasma concentrations (C_max_) were recorded as 23.45 ± 23.31, 31.36 ± 18.72, and 287.12 ± 253.07 ng/mL, respectively.

Following oral injection of tylvalosin in chickens, the plasma concentration–time curve was best described by a one-compartment open model. The secondary parameters are presented in Table 4. At doses of 5, 10, and 25 mg/kg, the drug was absorbed in chickens with absorption half-lives (t_1/2ka_) of 1.03 ± 1.14, 1.99 ± 0.92, and 1.05 ± 0.38 h, respectively, and elimination half-lives (t_1/2e_) of 2.40 ± 2.87, 2.04 ± 0.93, and 1.06 ± 0.38 h, respectively.

**Figure 3:**
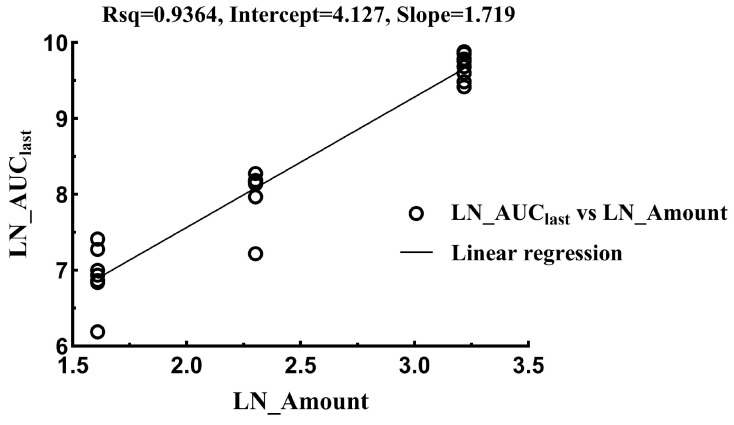
Regression analysis of relationship between LN_AUC_last_ and LN_Amount following intravenous administration.

**Figure 4:**
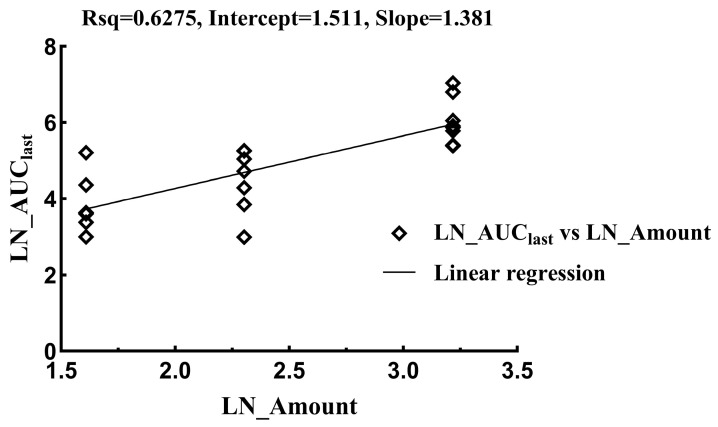
Regression analysis of relationship between LN_AUC_last_ and LN_Amount following oral administration.

The linear mixed-effect model was used to evaluate linearity in the range of 5–25 mg/kg, and the results are provided in Figure 3 and Figure 4 and Table 5 and Table 6. The relationship between the PK parameters (AUC_last_) and the dose was analyzed by using the power model, with the formula given in (1). If the 95% confidence interval for β falls within the reference range shown in Formula (2), then it is considered that the PK parameter is proportional to the dose. For the drug tylvalosin used in this study, where r = 5, the reference range for β is 0.86 to 1.14.

Ln(Parameter) = α + β × Ln(Dose)(1)

(2) $$\left[1+\frac{Ln\left(QL\right)}{Ln\left(r\right)},\;1+\frac{Ln\left(QU\right)}{Ln\left(r\right)}\right]$$

r: the ratio of the highest dose to the lowest dose.

QL: the lower equivalence limit, 0.8 (for AUC).

QU: the upper equivalence limit, 1.25 (for AUC).

The result indicates that the relationship between the PK parameters and the dose for the intravenous or oral administration of tylvalosin is not linear in the range of 5–25 mg/kg due to the *p*-value being less than 0.001 and the CI range being beyond the reference 0.86~1.14. Since there is no dose-proportional relationship for either intravenous or oral administration, the respective doses for each administration route were used to calculate the absolute bioavailability with Formulation (3). The absolute bioavailability values in the low, medium, and high oral groups were 5.92%, 3.56%, and 3.04%, respectively.

F = (AUC_po_)/(AUC_iv_) × 100% (3)

**Figure 5:**
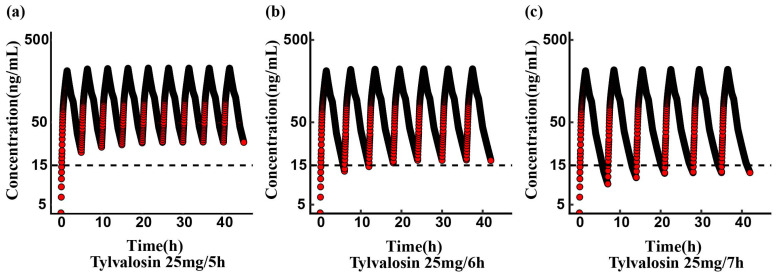
Computational simulations of tylvalosin concentration versus time at steady state were conducted for oral administration regimens of 25 mg/kg every 5 h (**a**), 6 h (**b**), and 7 h (**c**), respectively. Values above the dashed line indicate concentrations exceeding the therapeutic threshold of 15 ng/mL.

The study established different dosing intervals, and the simulation results indicated that dosing intervals longer than 6 h failed to achieve therapeutic concentrations (>15 ng/mL). The steady-state concentrations for the 25 mg/kg every 6-hour regimen are as follows: maximum concentration (C_max_), 222.91 ng/mL, and minimum concentration (C_min_), 17.22 ng/mL (Figure 5), and steady state was reached after four consecutive doses with a 6-hour interval.

## 4. Discussion

Tylvalosin, a novel veterinary macrolide antibiotic, has been proven effective in controlling respiratory infections in poultry and treating diseases associated with swine enteritis and pneumonia. In a pilot study, tylvalosin in chickens exhibited varying absorption profiles both between individuals and within the same individual when administered on separate occasions [21]. To further investigate the disposition of tylvalosin in chickens, a pharmacokinetic study was conducted by using oral and intravenous administration at three different doses.

In pharmacokinetic studies, the ADME (absorption, distribution, metabolism, and excretion) processes of a drug in the body can be described using either non-compartmental or compartmental models. In this study, after analyzing the semi-logarithmic plasma concentration–time curves, we found that the intravenous group exhibited characteristics of a two-compartment model, featuring two distinct linear phases: a rapid distribution phase followed by a slower elimination phase. In contrast, the oral group showed characteristics of a one-compartment model, with a single linear phase corresponding to the elimination phase. The use of compartmental modeling allows for a more detailed description of the drug’s complex distribution and elimination processes.

Following a single intravenous injection of tylvalosin in chickens, the plasma concentration–time curve displayed a biphasic pattern, best characterized by a two-compartment open model [13,15,22].

The distribution half-life (t_1/2α_) of tylvalosin across the three doses was approximately 0.12–0.13 h in our study. The data showed that tylvalosin was rapidly distributed, probably due to extensive tissue distribution. Similarly, at the same dosage, the distribution half-life (t_1/2α_) was reported as 0.153 h in broiler chickens [23] and 0.12 h in laying hens at a dose of 10 mg/kg body weight of tylosin [24].

Tylvalosin was rapidly eliminated, with an elimination half-life (t_1/2β_) of 0.63 h at a dose of 25 mg/kg body weight. This finding is consistent with previously reported values for tylvalosin in turkeys (0.79 h) [15] and tylosin in broiler chickens (0.52 h) [23]. Similarly, in laying hens, an elimination half-life of 0.61 h was observed at a dose of 10 mg/kg body weight of tylosin [24], closely aligning with our results (0.86 h).

The total clearance (CL = 2.24 L/h/kg) observed in the present study was slightly higher than the values reported for tylvalosin in turkeys (1.17 L/h/kg). Comparable clearance values of 1.50 L/h/kg and 0.95 L/h/kg were also reported for tylvalosin in turkeys and broiler chickens, respectively, following intravenous injection [15,25]. At a dose of 10 mg/kg, a higher clearance value of tylvalosin (4.28 L/h/kg) was recorded in this study, which is consistent with that reported for tylosin in laying hens (4.37 L/h/kg) [24].

In the present study, the total volume of distribution at steady state (Vss) at the dose of 25 mg/kg in chickens was determined to be 1.23 L/kg. This value is comparable to the Vss values observed for tylosin phosphate (1.09 L/kg) and tylosin tartrate (0.94 L/kg) in chickens [26] and exceeds the previously reported Vss of tylosin in broiler chickens (0.69 L/kg) [23]. The relatively low plasma protein binding rate of tylvalosin (13%) likely increases the proportion of free active drug available for distribution into extravascular compartments [13,15]. In contrast, tylosin’s higher plasma protein binding rate and lower cellular penetration result in lower Vss. As a weakly basic and highly lipophilic macrolide with a pKa of 7.6, tylvalosin facilitates passive diffusion into tissues at the plasma pH of 7.4. In low pH environments, such as inflamed areas, tylvalosin becomes ionized and exists in its charged form, which cannot easily cross cell membranes, causing it to become “trapped” in the tissues. This “ion-trapping effect” increases tylvalosin’s concentration and retention time in tissues [13,15,27,28]. Moreover, tylvalosin’s ability to penetrate and accumulate within specific cell types, such as gut epithelial cells, further boosts its tissue concentrations [29]. In our study, the total volume of distribution at steady state (Vss) decreased with the increase in the dose, possibly due to several factors. As the dose increases, tissue binding sites may become saturated, limiting the drug’s distribution into tissues and resulting in a greater proportion of the drug remaining in plasma. Furthermore, the ion-trapping effect may diminish at higher doses (e.g., when the tissue’s capacity to retain the drug approaches saturation), leading to more drugs being retained in plasma and a corresponding decrease in Vss.

In our study, the plasma concentration–time profiles of tylvalosin did not show parallel trends across the three dose levels. The AUC_last_ values at doses of 5, 10, and 25 mg/kg were 1.09, 3.18, and 16.25 h*µg/mL, respectively. As the dose of tylvalosin increased, AUC_last_ showed a disproportionate increase, which may be related to nonlinear distribution and nonlinear elimination following intravenous administration [30]. According to the above results, the saturation of metabolic enzymes and transporters is a critical factor underlying the nonlinearity observed at higher doses. Since macrolides are mainly metabolized by cytochrome P4503A (CYP3A) [29,31], as drug concentrations increase, these enzymes and transporters approach their maximum catalytic and transport capacities, leading to a reduction in clearance (CL) and the accumulation of the drug in the plasma. This saturation in metabolic and transport processes disrupts the proportional relationship between dose and key pharmacokinetic parameters, including CL, Vss, and AUC.

Following a single oral administration of tylvalosin (25 mg/kg body weight) in chickens, the drug reached a maximum concentration (C_max_) of 0.29 µg/mL at the T_max_ of 1.88 h post-administration. This C_max_ value is similar to that reported for tylvalosin in turkeys (0.64 µg/mL) [15] but lower than the C_max_ observed in chickens in another study (1.64 µg/mL) [32]. A.M. suggested that the lower C_max_ may be attributed to the presence of microbial flora in the crop, such as Lactobacillus species, which could inactivate macrolides [33].

The results of the present study demonstrate that tylvalosin was rapidly absorbed from the alimentary tract of chickens, as indicated by the short half-lives of absorption phase (t_1/2ka_ = 1.05 h) and absorption rate constant (K_a_ = 0.73/h). These results are similar to those obtained in turkeys (t_1/2ka_ = 0.96 h, K_a_ = 0.75/h) following oral administration at a dose of 25 mg/kg body weight. The pharmacokinetics of tylvalosin in chickens exhibited significant variability in absorption profiles, both between individuals and within the same individual.

Our findings show that the elimination half-life (t_1/2e_) of tylvalosin was 1.06 h, similar to the 0.96 h reported in turkeys and the 1 to 1.45 h observed in other poultry species [13,15]. This difference may be related to structural changes in tylvalosin compared with tylosin, specifically the addition of the isovaleryl group.

Following oral administration, the plasma concentration–time profile exhibited a non-parallel pattern, with a disproportionate increase in AUC_last_ (64.32, 113.28, and 493.83 h·ng/mL) as the dose increased. These findings suggest that the nonlinearity observed in tylvalosin’s pharmacokinetics may result from the saturation of metabolic enzymes, which leads to a decreased rate of drug clearance. Furthermore, when administered orally, tylvalosin might precipitate in its salt form within the digestive tract due to pH fluctuations, leading to reduced solubility and absorption efficiency. This effect is particularly pronounced at higher doses, where the solubility limit is more likely to be exceeded. Such factors could explain the low absolute bioavailability observed across all three doses, especially at the highest dose.

The measured C_max_ values of tylvalosin within 6 h after oral administration at the dose of 25 mg/kg in this study exceeded the reported minimum inhibitory concentrations (MICs) of tylvalosin against several MG (15–30 ng/mL) and MS (15 ng/mL) isolates from turkeys in Egypt [12]. According to recent research [34], the sensitivity patterns of 100 MG isolates to biocides and antibiotics were examined to elucidate the interactions between antimicrobial agents and resistance mechanisms. The results indicated that the MIC_50_ of tylvalosin was 62 ng/mL. Furthermore, compared with other macrolide antibiotics, tylvalosin exhibited the lowest MIC_90_ at 250 ng/mL (range 1–1000 ng/mL), highlighting its superior efficacy against MG.

This study simulated the multiple-dose regimen of tylvalosin (25 mg/kg) based on pharmacokinetic data from a single oral dose. The results indicate that a 6-hour dosing interval reaches a steady state after the fourth dose, while a 5-hour dosing interval may lead to drug accumulation in the body, thereby increasing the risk of side effects and promoting the development of drug resistance in pathogens. In poultry, controlling MG largely depends on antibiotics, which has led to significant challenges such as resistance development and potential cross-resistance to common antibiotics [35]. Therefore, rational dosing intervals and dosages are crucial for controlling the development of resistance. By optimizing dosing regimens (such as avoiding overuse and drug accumulation), the risk of resistance can be minimized. Furthermore, further research on the PK/PD (Pharmacokinetics and Pharmacodynamics) interactions of tylvalosin in vivo is essential for promoting the rational use of antibiotics and improving the sustainability of antimicrobial strategies in poultry production.

Additionally, we recognize that pharmacodynamic studies in lung and gut tissues are crucial for further elucidating tylvalosin’s drug distribution and effectiveness. Regarding the potential for drug precipitation in the gut, this is an important consideration. Factors such as local pH, enzymatic activity, and feed composition could influence tylvalosin’s solubility and absorption, thereby affecting its bioavailability and therapeutic efficacy. Investigating drug concentrations in gut tissues and luminal contents would help determine whether precipitation occurs and its potential impact on drug action. Furthermore, pharmacodynamic studies in lung tissues are essential for understanding tylvalosin’s distribution and retention in treating respiratory infections. The lungs are a primary target organ for tylvalosin in the treatment of swine pneumonia, and studying drug concentrations in this tissue would provide direct insights into its therapeutic potential. Therefore, we believe that future pharmacodynamic studies in lung and gut tissues would significantly enhance our understanding of tylvalosin’s distribution characteristics and its mechanisms of action in treating target infections. These studies would not only optimize dosing regimens but also provide a more robust scientific basis for its application across different animals.

However, further studies on enhancing the bioavailability of oral formulation are necessary. Relevant strategies may include the development of advanced drug delivery systems, such as lipid-based formulations or nanoparticle technologies, to enhance the solubility and dissolution of drugs [36,37,38]. Additionally, optimizing the choice of excipients, such as surfactants and absorption enhancers, can facilitate drug release and uptake in the gastrointestinal tract [39,40,41]. These approaches, supported by rigorous preclinical and clinical evaluations, could lead to a significant improvement in the oral bioavailability of tylvalosin and similar compounds.

## 5. Conclusions

In this study, we investigated the pharmacokinetic characteristics of tylvalosin at three different doses following both oral and intravenous administration in broiler chickens. The pharmacokinetic profiles demonstrated that tylvalosin is rapidly distributed and eliminated after intravenous administration, while it is quickly absorbed following oral administration. The observed non-parallel plasma concentration–time profiles and the disproportionate increase in AUC_last_ with doses ranging from 5 to 25 mg/kg indicate nonlinearity in the distribution of tylvalosin in broiler chickens. Based on the computational simulations from our study, a multiple-dose regimen of tylvalosin (25 mg/kg with a 6-h dosing interval) will reach a steady state after the fourth dose. Although an oral dose of 25 mg/kg of tylvalosin may provide effective coverage against MG and MS infections within 6 h, the absolute bioavailability of tylvalosin in chickens remains relatively low. Future research should focus on optimizing the formulation of tylvalosin to enhance its bioavailability and ensure more efficient therapeutic outcomes.

## Figures and Tables

**Table 1:** The major pharmacokinetic parameters of tylvalosin in chickens (mean ± SD, *n* = 8) after intravenous administration as determined with a non-compartmental analysis.

Parameter	Units	5 mg/kg	10 mg/kg	25 mg/kg
T_1/2λz_	h	5.98 ± 3.88	5.90 ± 1.89	6.78 ± 2.52
λz	1/h	0.15 ± 0.06	0.13 ± 0.04	0.11 ± 0.04
C_max_	ug/mL	2.05 ± 0.88	5.21 ± 1.71	25.44 ± 7.43
AUC_INF_obs_	h∗ug/mL	1.11 ± 0.40	3.26 ± 0.86	16.29 ± 2.67
AUC_last_	h∗ug/mL	1.09 ± 0.38	3.18 ± 0.86	16.25 ± 2.67
MRT_last_	h	1.56 ± 0.23	2.04 ± 0.38	0.82 ± 0.06
CL	L/h/kg	5.16 ± 2.34	3.42 ± 1.60	1.57 ± 0.27
Vd	L/kg	41.21 ± 23.56	29.54 ± 16.20	15.85 ± 7.82

Abbreviations: T_1/2λz_, elimination half-life; λz, elimination rate constant; C_max_, maximum whole-blood concentration; AUC_INF_obs_, area under concentration/time curve from 0 to infinity; AUC_last_, area under concentration/time curve from 0 to last point; MRT_last_, mean retention time; CL, total clearance; Vd, the apparent volume of distribution.

**Table 2:** The major pharmacokinetic parameters of tylvalosin in chickens (mean ± SD, *n* = 8) after intravenous administration as determined with a two-compartmental analysis.

Parameter	Units	5 mg/kg	10 mg/kg	25 mg/kg
A	ng/ml	3875.92 ± 2108.61	7463.25 ± 2975.44	34,392.08 ± 20,485.44
α	1/h	9.47 ± 5.16	7.35 ± 3.75	6.74 ± 2.91
t_1/2α_	h	0.13 ± 0.14	0.12 ± 0.06	0.12 ± 0.06
B	ng/ml	525.33 ± 393.52	1447.15 ± 824.28	8003.27 ± 3916.61
β	1/h	1.07 ± 0.67	0.91 ± 0.37	1.22 ± 0.44
t_1/2β_	h	1.83 ± 2.35	0.86 ± 0.30	0.63 ± 0.21
K_e_	1/h	4.68 ± 2.25	3.40 ± 1.21	3.64 ± 1.54
K_12_	1/h	3.61 ± 2.28	2.92 ± 2.10	1.96 ± 1.18
K_21_	1/h	2.25 ± 1.49	1.94 ± 0.94	2.35 ± 1.07
CL	L/h/kg	6.03 ± 3.26	4.28 ± 2.04	2.24 ± 0.37
Vc	L/kg	1.73 ± 1.37	1.42 ± 0.93	0.72 ± 0.30
V2	L/kg	3.24 ± 2.55	1.93 ± 1.14	0.51 ± 0.15
Vss	L/kg	4.97 ± 3.60	3.35 ± 1.99	1.23 ± 0.32

Abbreviations: A and B, zero-time intercepts of the biphasic disposition curve; α and β, hybrid rate constants representing the slopes of distribution and elimination phases, respectively; K_12_, first-order constant for transfer from the central to the peripheral compartment; K_21_, first-order constant for transfer from the peripheral to the central compartment; K_e_, elimination rate constant; t_1/2α_, distribution half-life; t_1/2β_, elimination half-life; Vss, the total volume of the distribution at steady state; Vc, the apparent volume of the central compartment; V2, the apparent volume of the peripheral compartment; CL, total clearance.

**Table 3:** The major pharmacokinetic parameters of tylvalosin in chickens (mean ± SD, *n* = 8) after oral administration as determined with a non-compartmental analysis.

Parameter	Units	5 mg/kg	10 mg/kg	25 mg/kg
T_1/2λz_	h	5.06 ± 3.13	4.19 ± 3.5	1.86 ± 0.83
λz	1/h	0.22 ± 0.17	0.24 ± 0.12	0.43 ± 0.15
C_max_	ng/mL	23.45 ± 23.31	31.36 ± 18.72	287.12 ± 253.07
AUC_INF_obs_	h*ng/mL	85.77 ± 73.15	136.7 ± 58.17	503.21 ± 341.91
AUC_last_	h*ng/mL	64.32 ± 61.52	113.28 ± 69.57	493.83 ± 337.16
Vd/F	mL/kg	1163.69 ± 1040.83	584.53 ± 618.78	177.12 ± 99.24
CL/F	mL/h/kg	172.93 ± 91.44	89.63 ± 49.72	67.39 ± 33.03
T_max_	h	2.17 ± 1.97	2.64 ± 0.95	1.88 ± 0.69
MRT_last_	h	3.51 ± 1.46	4.80 ± 2.23	2.67 ± 0.73

**Table 4:** The major pharmacokinetic parameters of tylvalosin in chickens (mean ± SD, *n* = 8) after oral administration as determined with a one-compartmental analysis.

Parameter	Units	5 mg/kg	10 mg/kg	25 mg/kg
K_a_	1/h	1.80 ± 1.77	0.42 ± 0.22	0.73 ± 0.23
t_1/2ka_	h	1.03 ± 1.14	1.99 ± 0.92	1.05 ± 0.38
Ke	1/h	0.63 ± 0.39	0.41 ± 0.21	0.72 ± 0.21
t_1/2e_	h	2.40 ± 2.87	2.04 ± 0.93	1.06 ± 0.38
V/F	L/kg	313.61 ± 281.03	325.33 ± 250.29	87.73 ± 42.80
CL/F	L/h/kg	122.67 ± 108.67	115.83 ± 84.01	59.39 ± 30.46

Abbreviations: Ka, absorption rate constant; K_e_, elimination rate constant; t_1/2ka_, half-lives of absorption phase; t_1/2e_, elimination half-life; V/F, apparent volume of distribution of fraction absorbed; CL/F, clearance of fraction absorbed.

**Table 5:** The results of the linear property for tylvalosin based on a linear mixed-effect model following intravenous administration.

Model	Slope Estimate	F-Stat	*p*-Value	Lower CI (95%)	Upper CI (95%)
Power model	1.72			1.51	1.93
One-way ANOVA		143.52	<0.001		

**Table 6:** The results of the linear property for tylvalosin based on a linear mixed-effect model following oral administration.

Models	Slope Estimate	F-Stat	*p*-Value	Lower CI (95%)	Upper CI (95%)
Power model	1.38			0.87	1.89
One-way ANOVA		16.23	<0.001		

## Data Availability

The data that support the study findings are available upon request and after authorization by the authors.

## References

[1] Feberwee A., de Wit S., Dijkman R. (2022). Clinical expression, epidemiology, and monitoring of Mycoplasma gallisepticum and Mycoplasma synoviae: An update. Avian Pathol..

[2] Feberwee A., Morrow C.J., Ghorashi S.A., Noormohammadi A.H., Landman W.J. (2009). Effect of a live Mycoplasma synoviae vaccine on the production of eggshell apex abnormalities induced by a M. synoviae infection preceded by an infection with infectious bronchitis virus D1466. Avian Pathol..

[3] Landman W.J. (2014). Is Mycoplasma synoviae outrunning Mycoplasma gallisepticum? A viewpoint from the Netherlands. Avian Pathol..

[4] Kreizinger Z., Grózner D., Sulyok K.M., Nilsson K., Hrivnák V., Benčina D., Gyuranecz M. (2017). Antibiotic susceptibility profiles of Mycoplasma synoviae strains originating from Central and Eastern Europe. BMC Vet. Res..

[5] Fiorentin L., Soncini R.A., da Costa J.L., Mores M.A., Trevisol I.M., Toda M., Vieira N.D. (2003). Apparent eradication of Mycoplasma synoviae in broiler breeders subjected to intensive antibiotic treatment directed to control Escherichia coli. Avian Pathol..

[6] Hong Y.H., Kwon J.S., Lee H.J., Song C.S., Lee S.W. (2015). Eradication of Mycoplasma synoviae from a multi-age broiler breeder farm using antibiotics therapy. Poult. Sci..

[7] Razin S., Hayflick L. (2010). Highlights of mycoplasma research--an historical perspective. Biologicals.

[8] Kursa O., Tomczyk G., Sieczkowska A., Kostka S., Sawicka-Durkalec A. (2024). Mycoplasma gallisepticum and Mycoplasma synoviae in Turkeys in Poland. Pathogens.

[9] Landman W.J., Mevius D.J., Veldman K.T., Feberwee A. (2008). In vitro antibiotic susceptibility of Dutch Mycoplasma synoviae field isolates originating from joint lesions and the respiratory tract of commercial poultry. Avian Pathol..

[10] Forrester C.A., Bradbury J.M., Dare C.M., Domangue R.J., Windsor H., Tasker J.B., Mockett A.P. (2011). Mycoplasma gallisepticum in pheasants and the efficacy of tylvalosin to treat the disease. Avian Pathol..

[11] Limpavithayakul K., Sasipreeyajan J., Pakpinyo S. (2023). Molecular characterization and antimicrobial susceptibility profiles of Thai Mycoplasma synoviae isolates. Sci. Rep..

[12] Abd El-Hamid M.I., Awad N.F.S., Hashem Y.M., Abdel-Rahman M.A., Abdelaziz A.M., Mohammed I.A.A., Abo-Shama U.H. (2019). In vitro evaluation of various antimicrobials against field mycoplasma gallisepticum and mycoplasma synoviae isolates in Egypt. Poult. Sci..

[13] Elbadawy M., Aboubakr M., Abugomaa A. (2019). Pharmacokinetics of Tylvalosin in Broiler Turkeys (Meleagris Gallopavo) After Single Intravenous and Oral Administration. Front. Vet. Sci..

[14] Huang G., Okabe M., Kahar P., Tsunekawa H., Park Y. (2001). Optimization of tylosin feeding rate profile in production of acetyl-isovaleryl tylosin (AIV) from tylosin by Streptomyces thermotolerans YN554. J. Biosci. Bioeng..

[15] Radi A.M. (2016). Pharmacokinetics and bioavailability of tylvalosin after oral, intramuscular and intravenous administration in turkeys. Int. J. Pharm. Pharm. Sci..

[16] Zhao Z., Tang X., Zhao X., Zhang M., Zhang W., Hou S., Yuan W., Zhang H., Shi L., Jia H. (2014). Tylvalosin exhibits anti-inflammatory property and attenuates acute lung injury in different models possibly through suppression of NF-κB activation. Biochem. Pharmacol..

[17] Guedes R.M., França S.A., Machado G.S., Blumer M.A., da Costa Cruz E.C. (2009). Use of tylvalosin-medicated feed to control porcine proliferative enteropathy. Vet. Rec..

[18] Hernandis V., Escudero E., Galecio J.S., Marín P. (2022). Quantification and Determination of Stability of Tylvalosin in Pig Plasma by Ultra-High Liquid Chromatography with Ultraviolet Detection. Animals.

[19] De Lorenzi G., Gherpelli Y., Luppi A., Pupillo G., Bassi P., Dottori M., Di Donato A., Merialdi G., Bonilauri P. (2024). In vitro susceptibility of Brachyspira hyodysenteriae strains isolated in pigs in northern Italy between 2005 and 2022. Res Vet Sci.

[20] Abu-Basha E.A., Bani Ismail Z., Idkaidek N.M., Hamzeh E. (2023). Comparison of pharmacokinetics of two tylvalosin oral formulations in broiler chickens. J. Vet. Pharmacol. Ther..

[21] Cerdá R.O., Giacoboni G.I., Xavier J.A., Sansalone P.L., Landoni M.F. (2002). In vitro antibiotic susceptibility of field isolates of Mycoplasma synoviae in Argentina. Avian Dis..

[22] Baggot J.D., Gingerich D.A. (1976). Pharmacokinetic interpretation of erythromycin and tylosin activity in serum after intravenous administration of a single dose to cows. Res. Vet. Sci..

[23] Kowalski C., Roliński Z., Zań R., Wawron W. (2002). Pharmacokinetics of tylosin in broiler chickens. Pol. J. Vet. Sci..

[24] Lina L. (2008). Pharmacokinetics of Acetylisovaleryltylosin Tartrate in Laying Hens. Master’s Thesis.

[25] Salman A.H., Youssef S., Ramadan A., Soliman A.M. (2016). Pharmacokinetics of tylvalosin in healthy and experimentally Mycoplasma gallisepticum infected broiler chickens. Int. J. PharmTech Res..

[26] Ji L.W., Dong L.L., Ji H., Feng X.W., Li D., Ding R.L., Jiang S.X. (2014). Comparative pharmacokinetics and bioavailability of tylosin tartrate and tylosin phosphate after a single oral and i.v. administration in chickens. J. Vet. Pharmacol. Ther..

[27] Fricke J.A., Clark C.R., Boison J.O., Chirino-Trejo M., Inglis T.E., Dowling P.M. (2008). Pharmacokinetics and tissue depletion of tilmicosin in turkeys. J. Vet. Pharmacol. Ther..

[28] Goudah A., Abo El Sooud K., Abd El-Aty A.M. (2004). Pharmacokinetics and tissue residue profiles of erythromycin in broiler chickens after different routes of administration. Dtsch. Tierarztl. Wochenschr..

[29] Stuart A.D., Brown T.D.K., Imrie G., Tasker J.B., Mockett A.P.A. (2007). Intra-cellular accumulation and trans-epithelial transport of Aivlosin, Tylosin and Tilmicosin. Pig J..

[30] Ludden T.M. (1991). Nonlinear pharmacokinetics: Clinical Implications. Clin. Pharmacokinet..

[31] Montesissa C., Capolongo F., Santi A., Biancotto G., Dacasto M. (2004). Metabolism of tilmicosin by rabbit liver microsomes and hepatocytes. Vet. J..

[32] Cerdá R.O., Petruccelli M., Piscopo M., Origlia J., Landoni M. (2010). Impact of the type of catheter on the absorption of tylvalosin (acetylvaleryltylosin) administered orally to broiler chickens. J. Vet. Pharmacol. Ther..

[33] Devriese L.A., Dutta G.N. (1984). Effects of erythromycin-inactivating Lactobacillus crop flora on blood levels of erythromycin given orally to chicks. J. Vet. Pharmacol. Ther..

[34] Kamal M.A., Salem H.M., Alhotan R.A., Hussein E.O., Galik B., Saleh A.A., Kaoud H.A. (2025). Unraveling Antimicrobial Resistance Dynamics in Mycoplasma gallisepticum: Insights Into Antibiotic and Disinfectant Interactions. Vet. Med. Sci..

[35] Gharaibeh S., Al-Rashdan M. (2011). Change in antimicrobial susceptibility of Mycoplasma gallisepticum field isolates. Vet. Microbiol..

[36] Lall A., Kamdem Tamo A., Doench I., David L., Nunes de Oliveira P., Gorzelanny C., Osorio-Madrazo A. (2020). Nanoparticles and Colloidal Hydrogels of Chitosan-Caseinate Polyelectrolyte Complexes for Drug-Controlled Release Applications. Int. J. Mol. Sci..

[37] Pangeni R., Panthi V.K., Yoon I.S., Park J.W. (2018). Preparation, Characterization, and In Vivo Evaluation of an Oral Multiple Nanoemulsive System for Co-Delivery of Pemetrexed and Quercetin. Pharmaceutics.

[38] Chen Z., Han S., Yang X., Xu L., Qi H., Hao G., Cao J., Liang Y., Ma Q., Zhang G. (2020). Overcoming Multiple Absorption Barrier for Insulin Oral Delivery Using Multifunctional Nanoparticles Based on Chitosan Derivatives and Hyaluronic Acid. Int. J. Nanomed..

[39] Ukai H., Imanishi A., Kaneda A., Kimura E., Koyama M., Morishita M., Katsumi H., Yamamoto A. (2020). Absorption-Enhancing Mechanisms of Capryol 90, a Novel Absorption Enhancer, for Improving the Intestinal Absorption of Poorly Absorbed Drugs: Contributions to Trans- or Para-Cellular Pathways. Pharm. Res..

[40] Guo X.H., Ding F., Lian X., Cui W., Li Z., Xing Y. (2021). The efficiency and mechanism of a new absorption enhancer, malic acid, for enhancing the oral bioavailability of docetaxel. Pharm. Dev. Technol..

[41] Lewis A.L., McEntee N., Holland J., Patel A. (2022). Development and approval of rybelsus (oral semaglutide): Ushering in a new era in peptide delivery. Drug Deliv. Transl. Res..

